# Supplementary material for: Projecting trends in the disease burden of adult edentulism in China between 2020 and 2030: a systematic study based on the global burden of disease
Source: Front Public Health. 2024 Apr 4;12:1367138. doi: 10.3389/fpubh.2024.1367138 (PMC11024259; doi:10.3389/fpubh.2024.1367138)
Supplement: Supplementary file 1 [file Data_Sheet_1.docx]

**Appendices**

TableS1 Projections of edentulism in China, 2020-2030 (1/100,000)

| year | Incidence（95%CI） | | Prevalence（95%CI） | | YLDs（95%CI） | |
| --- | --- | --- | --- | --- | --- | --- |
|  | Crude rate | Standardisation rate | Crude rate | Standardisation rate | Crude rate | Standardisation rate |
| 1990 | 208.32 (160.05-261.99) | 284.56 (223.75-352.49) | 2324.02 (1794.69-3022.13) | 3520.03 (2737.47-4463.25) | 63.92 (40.76-96.90) | 95.59 (61.29-142.54) |
| 1991 | 187.36 (145.30-235.34) | 257.00 (203.04-317.72) | 2057.87 (1601.32-2659.79) | 3101.10 (2426.08-3913.79) | 56.59 (36.17-85.70) | 84.15 (53.99-125.19) |
| 1992 | 169.78 (132.58-213.92) | 233.67 (185.59-287.69) | 1826.65 (1422.55-2349.32) | 2740.78 (2146.68-3439.76) | 50.21 (31.97-75.92) | 74.31 (47.59-110.33) |
| 1993 | 156.36 (121.87-197.80) | 215.38 (170.59-266.54) | 1644.85 (1284.62-2107.51) | 2456.40 (1918.69-3086.06) | 45.19 (28.74-68.28) | 66.54 (42.52-98.28) |
| 1994 | 148.06 (114.75-188.03) | 202.95 (160.78-251.30) | 1529.53 (1195.66-1955.14) | 2265.09 (1777.60-2846.93) | 41.99 (26.58-63.29) | 61.30 (39.05- 90.17) |
| 1995 | 145.64 (112.43-185.10) | 197.22 (155.42-244.93) | 1497.79 (1171.79-1910.97) | 2184.35 (1718.06-2773.17) | 41.10 (25.99-61.99) | 59.09 (37.77-86.72) |
| 1996 | 150.41(115.54-191.14) | 198.93 (156.70-247.98) | 1552.07 (1210.90-1976.45) | 2208.82 (1725.49-2807.51) | 42.58 (27.03-63.90) | 59.77 (38.17-87.80) |
| 1997 | 160.75 (122.39-205.33) | 206.18 (160.51- 257.62) | 1663.34 (1289.14-2123.86) | 2298.73 (1777.06-2929.99) | 45.62 (28.83-68.58) | 62.23 (39.55-92.06) |
| 1998 | 173.56 (131.76-222.03) | 215.81 (165.31- 270.97) | 1805.16 (1387.65-2309.83) | 2419.32 (1861.93-3098.77) | 49.50 (31.39-74.50) | 65.52 (41.72- 96.57) |
| 1999 | 185.76 (139.75-238.20) | 224.54 (171.68-282.50) | 1948.64 (1485.89-2497.03) | 2534.76 (1938.93-3251.04) | 53.43 (33.85-80.69) | 68.68 (43.73-102.00) |
| 2000 | 193.76 (145.37-246.64) | 229.08 (174.63-289.26) | 2058.56 (1560.32-2638.05) | 2608.85 (1986.52-3342.15) | 56.42 (35.65-85.45) | 70.71 (44.77-105.50) |
| 2001 | 201.42 (151.22-256.40) | 232.61 (177.68-292.94) | 2167.27 (1645.94-2776.04) | 2678.02 (2047.46-3421.20) | 59.41 (37.85-90.26) | 72.61 (46.20-108.48) |
| 2002 | 212.90 (161.51-269.80) | 239.22 (183.73-300.15) | 2316.07 (1778.71-2964.15) | 2786.26 (2140.68-3521.02) | 63.50 (40.54-96.34) | 75.58 (47.88-112.07) |
| 2003 | 226.22 (173.57-285.60) | 246.84 (191.90-307.94) | 2485.29 (1921.44-3174.97) | 2908.24 (2256.23-3663.72) | 68.18 (43.40-103.27) | 78.95 (50.33-117.08) |
| 2004 | 239.49 (185.40-301.30) | 253.40 (199.63-315.45) | 2655.52 (2060.76-3396.80) | 3018.47 (2343.69-3807.97) | 72.88 (46.40-109.88) | 81.98 (52.30-121.91) |
| 2005 | 249.48 (194.72-313.28) | 256.89 (202.64-319.02) | 2795.49 (2160.71-3583.00) | 3092.05 (2411.49-3900.85) | 76.73 (49.05-115.29) | 84.01 (53.43-125.22) |
| 2006 | 259.54 (202.75-326.15) | 259.83 (205.04- 322.65) | 2940.44 (2286.78-3760.54) | 3156.76 (2456.88-3981.99) | 80.74 (51.54-121.43) | 85.83 (54.56-127.97) |
| 2007 | 272.46(212.75-342.64) | 264.96 (207.90-329.24) | 3117.85 (2430.29-3981.75) | 3243.69 (2522.50-4092.00) | 85.65 (55.00-128.76) | 88.27 (56.19-131.48) |
| 2008 | 286.26 (223.31-360.29) | 270.54 (212.18-336.46) | 3303.04 (2564.43-4215.04) | 3332.13 (2588.27-4204.04) | 90.78 (58.33-136.47) | 90.77 (57.97-135.63) |
| 2009 | 299.40 (232.87-376.94) | 274.76 (214.95-342.10) | 3475.43 (2685.30-4434.83) | 3400.28 (2642.77-4296.05) | 95.56 (61.23-143.41) | 92.70 (59.05-138.79) |
| 2010 | 308.76 (239.60-388.24) | 275.91 (215.46-343.27) | 3600.18 (2778.49-4598.27) | 3427.00 (2658.96-4333.29) | 99.00 (63.00-148.75) | 93.47 (59.57-139.60) |
| 2011 | 306.47 (237.12-387.54) | 267.07 (208.88-333.20) | 3575.03 (2768.34-4551.35) | 3311.61 (2575.52-4180.95) | 98.29 (62.78-147.59) | 90.31 (57.63-134.92) |
| 2012 | 289.34 (223.96-364.77) | 247.43 (194.29-307.73) | 3360.95 (2618.63-4256.72) | 3037.76 (2376.28-3809.25) | 92.37 (59.33-138.95) | 82.80 (53.00-123.44) |
| 2013 | 265.89 (207.03-334.35) | 224.21 (178.07-277.53) | 3065.47 (2398.30-3865.47) | 2709.49 (2121.40-3369.28) | 84.22 (53.65-126.35) | 73.81 (47.24-109.62) |
| 2014 | 246.07 (191.66-309.65) | 204.59 (162.69-254.87) | 2813.40 (2199.19-3540.36) | 2430.14 (1908.46-3037.53) | 77.25 (48.81-115.79) | 66.16 (42.24-97.73) |
| 2015 | 239.89 (186.50-303.10) | 195.82 (155.40-244.28) | 2732.10 (2140.98-3431.34) | 2304.18 (1810.75-2872.02) | 74.98 (47.34-112.29) | 62.71 (40.00-92.13) |
| 2016 | 245.09 (190.44-309.92) | 194.92 (154.26-242.85) | 2792.50 (2187.15-3510.49) | 2292.11 (1806.18-2854.58) | 76.58 (48.70-114.40) | 62.36 (39.80-91.67) |
| 2017 | 251.20 (194.43-318.02) | 194.58 (154.01-242.16) | 2863.93 (2240.96-3609.08) | 2288.72(1802.00-2851.63) | 78.48 (50.05-117.33) | 62.26 (39.77-91.30) |
| 2018 | 295.41 (229.39-371.58) | 218.80 (173.76-269.75) | 3390.30 (2653.02-4313.06) | 2613.10 (2047.35-3259.17) | 92.82 (59.43-140.27) | 71.08 (45.58-106.03) |
| 2019 | 388.93 (298.29-491.58) | 272.15 (214.34-337.96) | 4512.78 (3485.26-5830.61) | 3327.48 (2589.33-4226.85) | 123.44 (79.31-186.25) | 90.52 (57.75-135.51) |
